# Research on WNN Modeling for Gold Price Forecasting Based on Improved Artificial Bee Colony Algorithm

**DOI:** 10.1155/2014/270658

**Published:** 2014-02-13

**Authors:** Bai Li

**Affiliations:** School of Control Science and Engineering, Zhejiang University, Hangzhou 310027, China

## Abstract

Gold price forecasting has been a hot issue in economics recently. In this work, wavelet neural network (WNN) combined with a novel artificial bee colony (ABC) algorithm is proposed for this gold price forecasting issue. In this improved algorithm, the conventional roulette selection strategy is discarded. Besides, the convergence statuses in a previous cycle of iteration are fully utilized as feedback messages to manipulate the searching intensity in a subsequent cycle. Experimental results confirm that this new algorithm converges faster than the conventional ABC when tested on some classical benchmark functions and is effective to improve modeling capacity of WNN regarding the gold price forecasting scheme.

## 1. Introduction

Since ancient times, gold has been recognized as a symbol of wealth and a frontierless currency that can be easily exchanged among different monetary systems [1, 2]. In recent decades, gold has gradually become a popular nonmonetary tool in the financial market, which is characterized by high-yield and high-risk. Gold price is partly regarded as a reflection of investors' expectations and the world's economic trends. Therefore, gold price forecasting is a vital issue in economics. At the same time, it is noted that, during the financial crisis in 2008 and early 2009, the global gold price has increased by 6% on average, while many mineral prices have dropped by 40% approximately [3]. In this sense, the gold price behavior differs from that of most other mineral commodities, making the forecasting scheme even more challenging.

Regarding the prediction of gold price, like the prediction of any other macroeconomic indexes, research efforts have been focused on the neural network (NN) approaches [4]. A neural network is known as a mathematical model consisting of interconnected groups of artificial neurons and processing information based on a connectionist approach to computation [5, 6]. In most cases, neural networks are adaptive systems that can alter the internal structures according to the external information. Ever since McCulloch and Pitts pioneering work [7], artificial models, such as back-propagation neural network (BP-NN) [8], radial basis function neural network (RBF-NN) [9], wavelet neural network (WNN) [10], Kohonen neural network [11], and Hopfield neural network [12] have been proposed and investigated. Among all these methods, WNN has shown its advantages in regression accuracy and fault-tolerant ability due to the adoption of wavelet transform. It has been confirmed that the WNN model is the optimal approximator for functions of one variable [13].

Large numbers of methods have been available to optimize WNN models, among which the gradient descent method (GDM) and the least square method (LS) are undoubtedly the most popular ones [14]. However, such conventional methods cannot help to optimize efficiently or globally in terms of some complicated WNN models. In other words, when the parameters in a WNN model are large in number or when the training scheme is complicated, these deterministic optimization methods are not as efficient as they are expected. Therefore, researchers have gradually shifted their interests towards some intelligent algorithms to optimize WNN models [15].

Intelligent algorithms have been well studied in recent decades, among which artificial bee colony (ABC) algorithm is a famous example. It is motivated by the foraging behavior of bee swarms, in which both local exploitation and global exploration are implemented in each iteration [16]. Applications and developments have been made for ABC in different ways [17–23]. However, viewing the improvements has ever been made for the conventional ABC; to the best of my knowledge, attention has seldom been paid to fully utilizing the convergence messages hiding in the iteration system. In this paper, internal-feedback ABC (IF-ABC) is applied for WNN parameter optimization when training a gold price prediction model. In this new algorithm, invalid trial time is taken as an index to reflect the internal status and then to manipulate the exploration/exploitation intensity. At this point, the author believes that, apart from the objective function values, messages that reflect the convergence status should be made full use of so as to direct the subsequent searching cycles [24].

As for gold price forecasting, neural network methods such as RBF-NN [25], BP-NN [26], and WNN [27] have been studied. This work provides an intensive research to evaluate the performance of IF-ABC when training the WNN models, in comparison with the conventional ABC algorithm.

The remainder of this paper is organized as follows. In Section 2, principle of the WNN model is briefly introduced. In Section 3, principles of ABC and IF-ABC are introduced in detail.  Section 4 validates the effectiveness of IF-ABC by means of some classical benchmark functions. Then, IF-ABC is applied to optimize the WNN models for the gold price forecasting scheme. Simulation results are released in Section 5, together with some discussions. The conclusion is drawn in the last section.

## 2. Principle of Wavelet Neural Network Model

WNN is a feed-forward neural network combined with the wavelet transform theory [15]. In such a framework, the wavelet space is regarded as a feature space, where features are extracted by weighting the interior states of the input signals. Compared with other NN models, WNN possesses higher prediction accuracy and better fault tolerance to meet the uncertainty, nonlinearity, and complexity in real-world systems [28].

**Figure 1:**
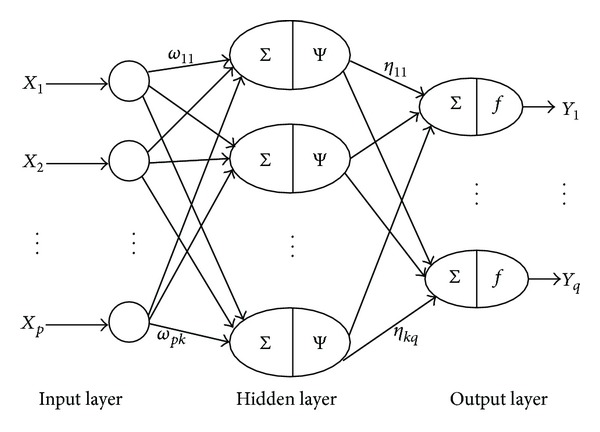
Basic WNN model structure.

The basic structure of a WNN model is illustrated in Figure 1, where *X*_*i*_  (*i* = 1,…, *p*) denotes the *i*th input, *Y*_*j*_  (*j* = 1,…, *q*) denotes the *j*th output, *ω*_*ij*_  (*i* = 1,…, *p*; *j* = 1,…, *k*) refers to the connection weight between the *i*th input node and the *j*th hidden node, *η*_*ij*_  (*i* = 1,…, *k*; *j* = 1,…, *q*) refers to the connection weight between the *i*th hidden node and the *j*th output node, *p* represents the number of input nodes, *k* represents the number of hidden nodes, and *q* represents the number of output nodes. Ψ(·) and *f*(·) stand for the *Morlet* and *Sigmoid* functions, which are defined in (1) and (2), respectively [14]:

(1) $$\begin{array}{c} \Psi \left(x\right)=\cos\left(1.75\cdot x\right)\cdot \exp\left(\frac{-x^{2}}{2}\right), \end{array}$$

(2) $$\begin{array}{c} f\left(x\right)=\frac{1}{1+\exp\left(-x\right)}. \end{array}$$

Each weighted sum of the input components ∑_*m*=1_^*p*^(*ω*_*mn*_ · *X*_*m*_) is mapped into the feature space by the dilation and translation procedures, yielding Ψ((∑_*m*=1_^*p*^(*ω*_*mn*_ · *X*_*m*_ − *b*_*j*_))/*a*_*j*_). Here, *a*_*j*_ and *b*_*j*_, respectively, denote the dilation factor and translation factor. The output *Y*_*i*_ is given as

(3) $$\begin{array}{c} Y_{i}=f\left(\overset{k}{\underset{n=1}{\sum }}\left[\eta_{ni}\cdot \frac{1}{\sqrt{a_{n}}}\Psi \left(\frac{\sum_{m=1}^{p}\omega_{mn}\cdot X_{m}-b_{n}}{a_{n}}\right)\right]\right). \end{array}$$

The training process of WNN is considered an estimation process to optimize the parameter set Θ = {*ω*_11_,…, *ω*_*pk*_, *η*_11_,…, *η*_*kq*_, *a*_1_,…, *a*_*k*_, *b*_1_,…, *b*_*k*_} as mentioned above. RMSE (i.e., root mean square error) that reflects WNN modeling accuracy is defined in (4). Obviously, the smaller the forecasting error is, the better the model will be:

(4) $$\begin{array}{c} \text{RMSE}\left(\Theta \right)=\sqrt{\frac{1}{S}\overset{S}{\underset{i=1}{\sum }}{||Y^{i}-\text{true}Y^{i}||}^{2}}, \end{array}$$

where true*Y* ^*i*^  (*i* = 1,…, *S*) stands for the *i*th output sample, *Y* ^*i*^  (*i* = 1,…, *S*) stands for the corresponding output value computed by WNN, and *S* denotes the number of training samples.

## 3. Principles of ABC Relevant Algorithms

### 3.1. Review of Conventional ABC

ABC is a swarm intelligence-based optimization algorithm inspired by the forging behavior of bees [16]. In this algorithm, three kinds of bees, namely, the employed bees, the onlooker bees, and the scout bees, cooperate to search for the very optimal nectar source in the space [23].

At the beginning, an initial population is randomly generated, which contains as many as *SN* food sources (i.e., *SN* feasible solutions) using (5). In this equation, each solution **X**_*i*_ = (*x*_*i*_^1^, *x*_*i*_^2^,…, *x*_*i*_^*D*^) is a *D*-dimensional vector, **X**_max⁡_ and **X**_min⁡_ stand for the constraints of the optimization problem, and rand(0,1) stands for a random number in the range (0,1) obeying the uniform distribution. Note that the variables involved in this section are not relevant to those emerged in Section 2:

(5) Xi⟵Xmin⁡+rand(0,1)·(Xmax⁡−Xmin⁡),i=1,2,…,SN.

Afterwards, the iteration process starts. Generally, in each cycle of iteration, as many as *SN* employed bees search globally, and then *SN* onlooker bees search locally for the “qualified” employed bees. Here, the qualification standard concerns the roulette selection strategy and will be introduced later.

In detail, each employed bee utilizes the position of its one randomly chosen companion so as to generate a new searching position. Here, only one (randomly chosen) element in the vector **X** needs to be changed. For instance, when the *i*th employed bee utilizes position of the *k*th companion in the *j*th element, the involved element is changed according to

(6) xij⟵xij+rand(−1,1)·(xkj−xij),j≠k, j,k∈[1,D]∩ℤ, i∈[1,SN]∩ℤ.

Afterwards, the greedy selection procedure is implemented. If the new position updated by (6) is better (i.e., if the corresponding objective function value is lower), the previous position is discarded; otherwise, the employed bee remains at the previous position. When all the *SN* employed bees complete the searching procedure mentioned above, an index *P* is calculated as the qualification measurement for the employed bees using

(7) $$\begin{array}{c} P\left(i\right)=\frac{\text{fitness}\left(i\right)}{\sum_{j=1}^{SN}\text{fitness}\left(j\right)},\\ \text{fitness}\left(i\right)=\left\{\begin{array}{cc} \frac{1}{1+\text{obj}\left(X_{i}\right)} & \text{if}\;\;\text{obj}\left(X_{i}\right)\geq 0\\ 1+\text{abs}\left(\text{obj}\left(X_{i}\right)\right) & \text{if}\;\;\text{obj}\left(X_{i}\right)<0 \end{array}\right\}, \end{array}$$

where obj(·) stands for the objective function and fitness(·) stands for a conventionally defined fitness function. Each onlooker bee needs to search for an employed bee using (6). In this case, *x*_*k*_^*j*^ stands for the corresponding element of the selected employed bee and *x*_*i*_^*j*^ denotes that of the *i*th onlooker bee. Again, the greedy selection procedure is then implemented.

The selection principle for the qualified employed bees concerns the roulette selection strategy. If *P*_1_ ≥ rand(0,1), the first employed bee is chosen for the specific onlooker bee; otherwise, comparison between *P*_2_ and rand(0,1) is carried on. If every *P*_*i*_ happens to be smaller than rand(0,1), such process will go over again until one employed bee satisfies the condition. In this way, each of the *SN* onlooker bees determines the corresponding employed bee to follow.

During each cycle of iteration, once the *i*th employed bee or an onlooker bee which searches for the *i*th employed bee finds a better position in the crossover procedure, the parameter trial(*i*) is directly reset to zero; otherwise, it adds one. In this sense, trial is regarded as a counter memorizing the invalid searching times for the *i*th employed bee. Before a new cycle of iteration starts, it is necessary to check whether any trial(*i*) exceeds a certain threshold Limit. If trail(*i*) > Limit, trial(*i*) will be directly reset to zero. A scout bee with a randomly initialized position in the food source utilizing (5) will take the place. It should be noted that one scout bee at most is allowed to emerge in each cycle of iteration.

### 3.2. Principle of IF-ABC

The author and some companions proposed IF-ABC originally in the previous literature [24, 29]. But this algorithm is slightly modified when presented in this work, aiming to make it more efficient.

At first, all the employed bees are randomly sent out to explore in the nectar source space (i.e., the feasible solution space) following (5). Afterwards, the iteration process gets started.

In each cycle of iteration, an employed bee exchanges information with its (randomly selected) companions. Different from that in ABC, the crossover procedure should involve as many as trial(*i*) elements in the position of the *i*th employed bee (see (8)), where trial(*i*) ∈ [1, *D*]∩*ℤ* is an issue to be discussed later:

(8) xij←xmj+rand  (−1,1)·(xkj−xij),m,j,k∈[1,D]∩ℤ, i∈[1,SN]∩ℤ, j≠k.

This equation is slightly different from (6), aiming to promote swarm diversity during the global exploration procedure. Then, the greedy selection procedure is conducted so as to select better position.

Afterwards, the onlookers carry on the searching process. In the IF-ABC, each of the employed bees is given a chance to be followed by an onlooker regardless of the fact that they are “qualified” or not, pursuing to bring about more chances (i.e., more dynamics and diversity) for evolution and to fight against premature convergence as well. In IF-ABC, a new idea is introduced to evaluate the qualification of a bee.

Now that the roulette selection strategy is discarded in IF-ABC; then the onlookers directly choose their corresponding employed bees to search locally using (9), where the companion **X**_*k*_ and the element item *j* are randomly selected. Afterwards, the greedy selection is implemented:

(9) xij←xij+γ(i)·rand(−1,1)·(xkj−xij),j≠k, j,k∈[1,D]∩ℤ, i∈[1,SN]∩ℤ,

where

(10) $$\begin{array}{c} \gamma \left(i\right)=\exp\left\{-\left[\text{trial}\left(i\right)-1\right]\cdot \frac{\ln10}{D-1}\right\}. \end{array}$$

For each of the employed bees, together with the corresponding onlookers, the parameter trial represents the number of inefficient searching times before even one better position is derived. If the *i*th employed bee or the *i*th onlooker bee finds a better position, trial(*i*) is directly reset to 1; otherwise, it adds 1. If trial(*i*) is greater than *D*, the current *i*th position **X**_*i*_ should be replaced by a reinitialized position using (5).

Since 1 ≤ trial(*i*) ≤ *D*, it is expected that as many as trial(*i*) out of the *D* elements in a candidate feasible solution involve in the exploration process. But when it comes to the onlooker bees, only one element is changed, because it is believed that multicrossover process contributes little to local search ability. Note that a convergence factor *γ*(*i*) appears in (9), which is carefully designed to manipulate the exploitation accuracy according to the current convergence status of the *i*th employed bee. As shown in (10), *γ*(*i*) decreases exponentially to 0.1 as trial(*i*) gradually approaches *D*. Here, 0.1 is a user-specified lower boundary of convergence scale, but the selection of such constant can be flexible according to the users. In this sense, the exploitation around one certain employed bee is gradually intensified before it is eventually discarded by means of reinitialization (when trial exceeds *D*).

To briefly conclude, trial in IF-ABC works to manipulate the searching intensity in local exploitation and to determine the searching scale in global exploration. In the author's viewpoint, convergence performances of the bees are measured not only by the corresponding objective function values but also by the facts whether they are better than the previous one. Such change intends to provide more possibilities for the so-called unqualified employed bees to be exploited locally by onlooker bees.

The pseudocode of IF-ABC for constrained optimization problems is given in Algorithm 1. MCN denotes the predefined maximum cycles of iteration.

## 4. Effectiveness Validation of IF-ABC for Numerical Optimization

In this section, ABC and IF-ABC are tested on a number of classical benchmark functions [30]. The concerned functions are listed in Table 1, together with the pre-defined optimization domains, optimums, and optimal solutions. In this table, dim⁡ stands for the dimension of feasible solutions. *f*1 is unimodal and *f*2, *f*3, *f*4, and *f*5 are multimodal.

**Figure 2:**
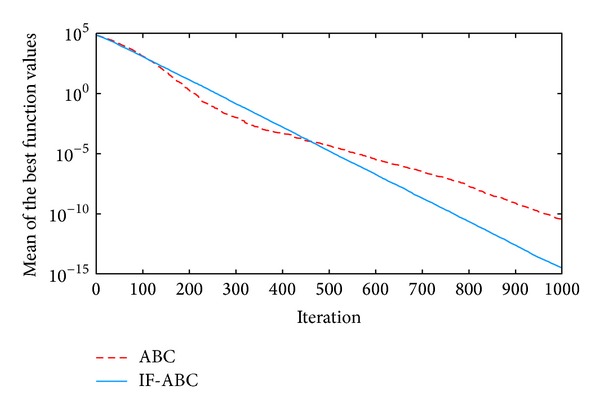
Comparative simulations for minimization of *f*1  (dim⁡ = 30).

**Figure 3:**
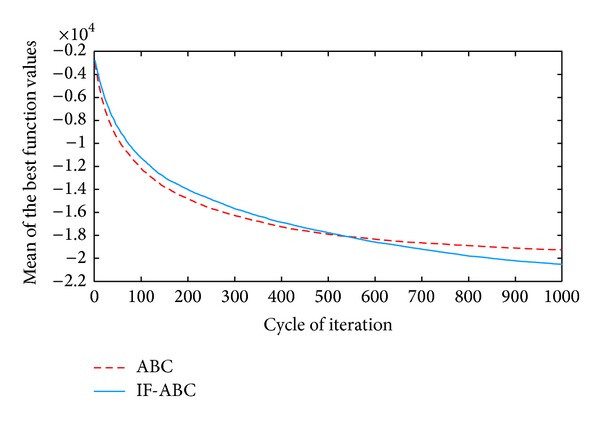
Comparative simulations for minimization of *f*2  (dim⁡ = 50).

**Figure 4:**
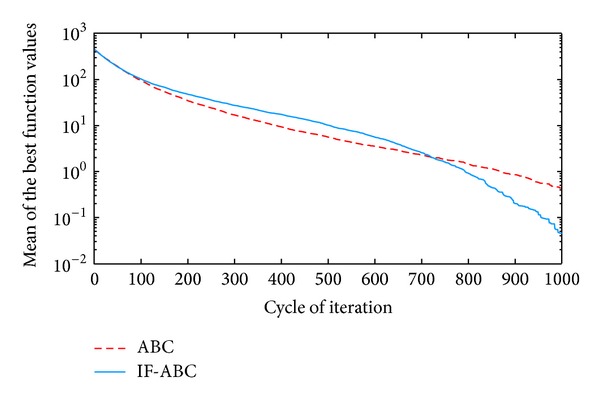
Comparative simulations for minimization of *f*3  (dim⁡ = 30).

**Figure 5:**
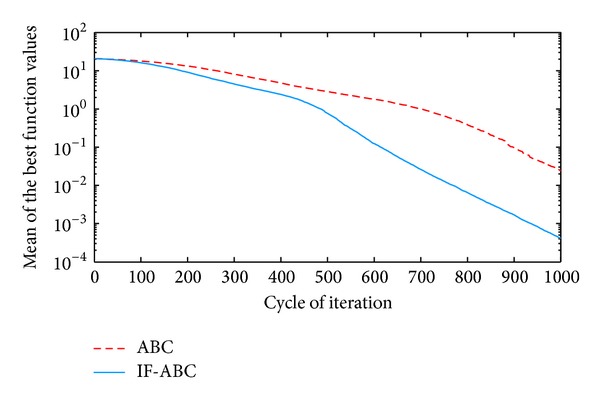
Comparative simulations for minimization of *f*4  (dim⁡ = 50).

**Figure 6:**
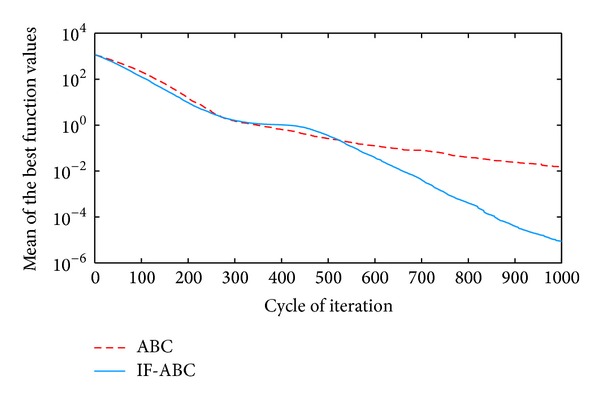
Comparative simulations for minimization of *f*5  (dim⁡ = 50).

All the simulations were implemented in MATLAB R2010a and executed on an Intel Core 2 Due CPU with 2 GB RAM running at 2.53 GHz. Each kind of experiment repeated itself 50 times with different random seeds. The maximum cycle number MCN is set to 1000 for all the cases involved in this section, the swarm population (i.e., 2 · *SN*) is constantly set to 40, and Limit is set to 200. Two indexes that reflect the convergence performances (i.e., the mean and standard deviation of benchmark function values) are listed in Table 2. Figures 2, 3, 4, 5, and 6 illustrate some typical simulation results to illustrate the significant advantage of IF-ABC.

As can be seen in Figures 2–6, the iteration process converges slower when using IF-ABC than it does when using ABC in the early cycles of iteration. But IF-ABC makes it catch up and be surpassed later. Initially, it is generally easy to evolve, regardless of the differences in algorithms. In other words, when more than one element of a feasible solution is involved in the crossover procedure, it does not necessarily lead to a better result in comparison with the case in which only one element is involved. However, as the iteration moves on, the internal feedback strategy begins to take effort. Therefore, it is believed that IF-ABC sacrifices part of its initial convergence capability for dynamics and diversity in the bee swarms. A complete comparison concerning these two algorithms is listed in Table 2, where IF-ABC performs far better (within 1000 cycles of iteration) in most of the cases.

The author noticed that many remedies for the conventional ABC come from the outside world (e.g., [18, 19]), ignoring utilizing the convergence status inside the iteration system. In this sense, IF-ABC intends to emphasize and advocate the great importance of fully utilizing internal status as feedback messages. In the event that IF-ABC really performs not so good as some existing X-ABCs, it does not mean that the internal feedback strategy is of no use. Therefore, the author preliminarily compared IF-ABC with ABC in all the experiments and simulations of this work.

## 5. Simulations for Gold Price Forecasting Scheme

**Figure 7:**
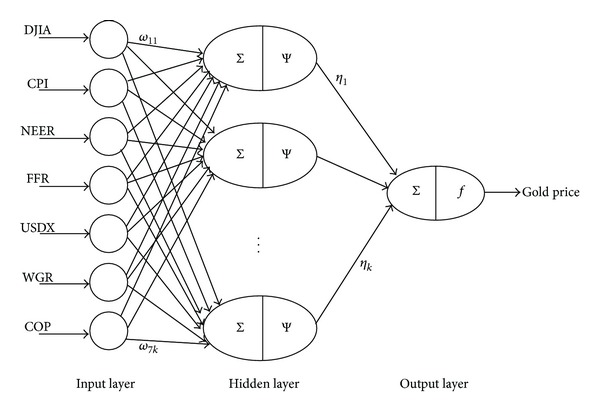
WNN model structure for long-term gold price forecasting.

**Figure 8:**
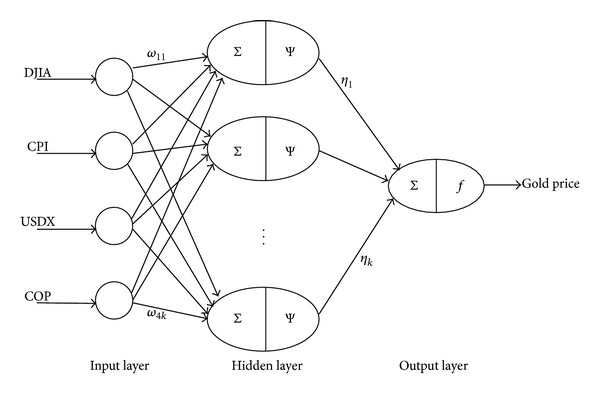
WNN model structure for short-term gold price forecasting.

The quantities of supply and demand, the prosperity of economics, and the environment of international politics mainly affect the gold price or may be regarded as good reflections of gold price in the future [3, 31, 32]. In this work, seven macroeconomic indexes are considered principal reflections of the long-term gold price in the future (i.e., gold price in the next year), namely, Dow Jones Industrial Average Index (DJIA), Consumer Price Index (CPI), US dollar nominal effective exchange rate (NEER), US federal funds rate (FFR), US dollar index (USDX), the world's gold reserves (WGR), and the world's crude oil price (COP) [33, 34]. The long-term prediction network structure is demonstratively given in Figure 7. In this case, four sensitive-about-time macroeconomic indexes are taken as principal reflections of the short-term gold price in the future (i.e., gold price in the next month), namely, DJIA, CPI, USDX, and COP. Then, the short-term prediction network structure is demonstratively given in Figure 8. Relevant historical data were collected from IHS Global Insight Inc (see http://www.ihs.com/index.aspx), website of the US Department of Labor (see http://www.bls.gov), website of the US Federal Reserve (see http://www.federalreserve.gov), and website of the International Monetary Fund (see http://www.imf.org/external/index.htm).

Each single type of experiment was repeated 50 times with randomly initialized conditions so as to guarantee the significant initial differences in statistics. It is set that *SN* = 20 and Limit = 200. All the connection weights in WNN (i.e., *ω* and *η*) range from 0 to 1, any *a* ranges from 0.0001 to 10, and any *b* ranges from −1 to 1. The determination of hidden layer node number *k* is theoretically unavailable. In general, if *k* is too large, the overfitting trouble inevitably occurs, and, conversely, if *k* is too small, the derived model will reflect anything but the true facts. In this work, *k* is selected using the following equation:

(11) $$\begin{array}{c} k=\sqrt{p\cdot q+1.6799\cdot p+0.9298\cdot q}, \end{array}$$

where *p* and *q* denote the number of input and output layer nodes, respectively [28]. Besides, all data put into the WNN model (i.e., economic indexes and the corresponding gold prices) should be linearly standardized in the range [0,1] as a preprocessing step, and the results worked out by WNN (i.e., the predicted future gold prices) involve an inverse process.

**Figure 9:**
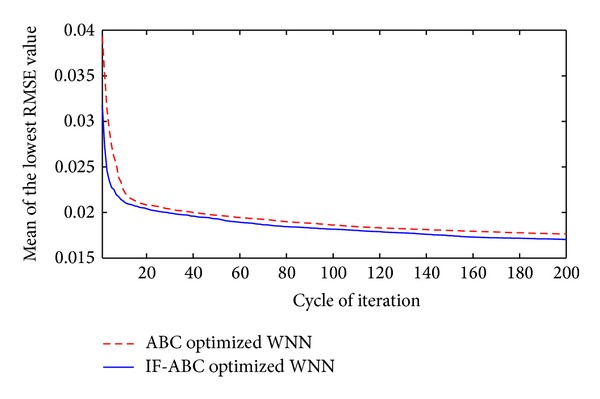
Comparative convergence curves when optimizing short-term WNN prediction models utilizing historical data of past 60 months.

**Figure 10:**
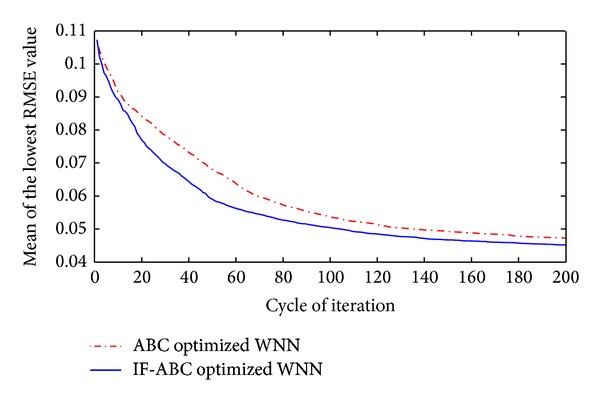
Comparative convergence curves when optimizing short-term WNN prediction models utilizing historical data of past 80 months.

**Figure 11:**
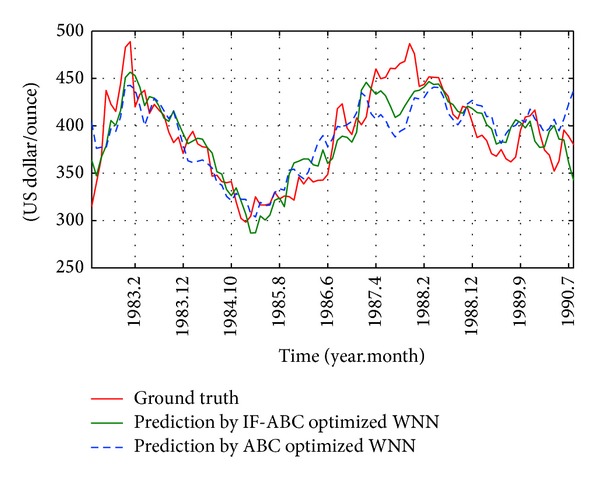
Monthly historical and future forecasting trends of gold price from April 1982 to August 1990 (case 1 in short-term).

**Figure 12:**
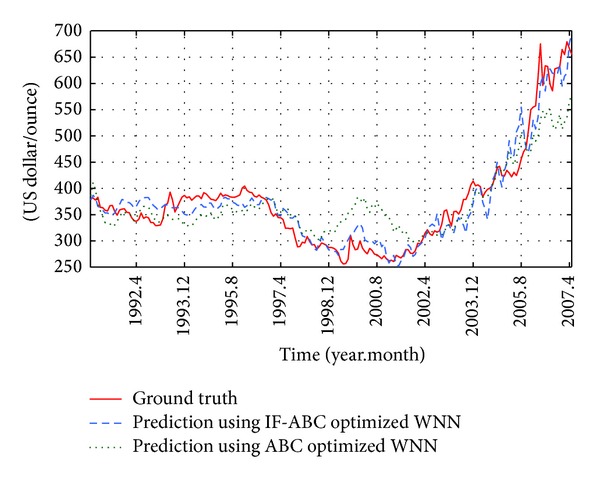
Monthly historical and future forecasting trends of gold price from September 1990 to April 2007 (case 2 in short-term).

First, two cases were studied to compare the convergence performances of IF-ABC and ABC when optimizing a short-term forecasting model. In the first case, the prediction model was trained using four macroeconomic indexes from April 1982 to August 1985 (as long as 60 months), and the optimized WNN model was tested on the data in each of the coming 40 months (i.e., from September 1985 to August 1990). In the second case, the prediction model was trained using four macroeconomic indexes from September 1990 to April 1997 (as long as 80 months), and the optimized WNN model was tested on the data in each of the coming 120 months (i.e., from May 1997 to April 2007). The comparative convergence curves are illustrated in Figures 9 and 10. As can be seen in the following Figures 11 and 12, the trend of gold price predictions derived by the IF-ABC-WNN model is closer to the actual gold price data.

**Figure 13:**
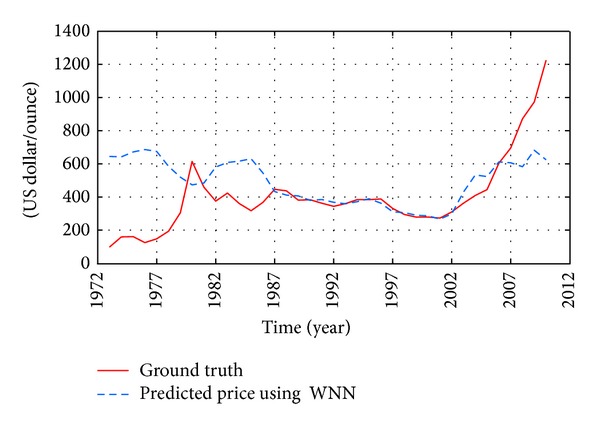
Yearly historical and future forecasting trends of gold price from 1973 to 2011 (a case in long-term).

In the long-term, such training methodologies are ineffective or invalid, since the principle how the macroeconomic indexes affect the gold price may be varying significantly. To confirm this point of view, an experiment was carried out in this work as well (see Figure 13). In this example, annual macroeconomic indexes from 1987 to 2000 are regarded as the training data. The derived WNN model is tested by forecasting the gold price trends from 1973 to 1986 and from 2001 to 2011. Figure 13 clearly depicts that the trained model only fits the actual gold prices well from 1987 to 2002. That is to say, the generalization ability of WNN is too weak to forecast the long-term gold price.

## 6. Conclusion

In this work, a modified version of ABC named IF-ABC is applied to optimize the WNN model in the gold price forecasting scheme. Series of numerical experiments confirm that IF-ABC is more effective than conventional ABC in the capability to train WNN models.

IF-ABC is applied in this work to advocate the viewpoint that, apart from the quality of a nectar source (i.e., the objective function value), the true convergence efficiency may also be reflected by the fact that whether a bee does find a position better than the previous one it stays at. The author believes that the internal feedback strategy in the IF-ABC algorithm may be applied to modify some other swarm intelligence algorithms.

Besides, further investigations into the relationship between the gold price and other key influencing variables, especially in the long-term, will be the future work.

## Figures and Tables

**Algorithm 1:**
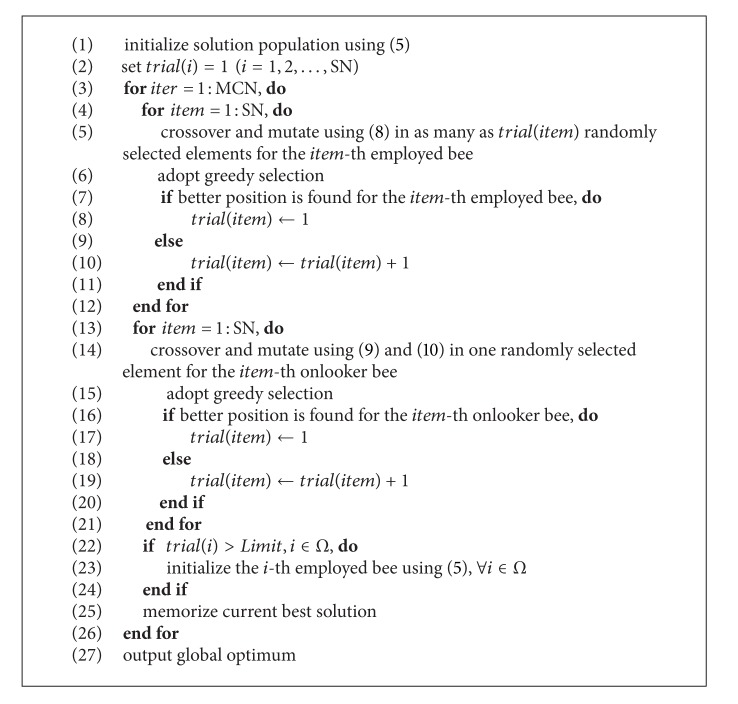


**Table 1:** Benchmark functions together with optimums and optimal solutions.

Test function	Ranges	Optimum	Optimal solution
$f_{1}\left(\vec{x}\right)=\sum_{i=1}^{\dim}{\left(x_{i}\right)}^{2}$	[−100,100]^dim⁡^	0	[0,0,…, 0]^dim⁡^
$f_{2}(\vec{x})=-\sum_{i=1}^{\dim}x_{i}\sin(\sqrt{\left|x_{i}\right|})$	[−500,500]^dim⁡^	−418.9829 · dim⁡	[420.96,420.96,…, 420.96]^dim⁡^
$f_{3}(\vec{x})=\sum_{i=1}^{\dim}\left[{\left(x_{i}-1\right)}^{2}-10\cos\left(2\pi \left(x_{i}-1\right)\right)+10\right]$	[−5.12,5.12]^dim⁡^	0	[1,1,…, 1]^dim⁡^
$\begin{array}{cc} f_{4}\left(\vec{x}\right)= & -20\exp\left(-0.2\sqrt{\frac{1}{\dim}\overset{\dim}{\underset{i=1}{\sum }}x_{i}}\right)\\ & -\exp\left(\frac{1}{\dim}\overset{\dim}{\underset{i=1}{\sum }}\cos\left(2\pi x_{i}\right)\right)+20+e \end{array}$	[−32,32]^dim⁡^	0	[0,0,…, 0]^dim⁡^
$f_{5}(\vec{x})=\frac{1}{4000}\sum_{i=1}^{\dim}{\left(x_{i}\right)}^{2}-\prod_{i=1}^{\dim}\cos\left(\frac{x_{i}}{\sqrt{i}}\right)+1$	[−600,600]^dim⁡^	0	[0,0,…, 0]^dim⁡^

**Table 2:** Mean and standard deviations of benchmark function values.

Test function	Dim	ABC		IF-ABC	
		Mean	S.D.	Mean	S.D.
*f*_1_	20	3.91095 × 10^−16^	8.20298 × 10^−17^	9.35983 × 10^−16^	5.51436 × 10^−16^
	30	3.60481 × 10^−11^	5.46368 × 10^−11^	3.36243 × 10^−15^	1.52689 × 10^−15^
	50	5.12689 × 10^−6^	7.57033 × 10^−6^	3.88046 × 10^−7^	1.43985 × 10^−7^

*f*_2_	20	−8296.94	71.2099	−**8374.8**	**23.4352**
	30	−12024.2	161.724	−**12543.4**	**60.0115**
	50	−19268.7	**207.685**	−**20528.7**	215.453

*f*_3_	20	1.68276 × 10^−7^	7.51168 × 10^−7^	9.18874 × 10^−12^	4.64979 × 10^−11^
	30	0.437308	0.605332	**0.0454379**	**0.21805**
	50	**6.73436**	**2.3812**	10.7746	2.77952

*f*_4_	20	2.3339 × 10^−9^	1.87748 × 10^−9^	6.05063 × 10^−13^	2.56131 × 10^−13^
	30	2.22333 × 10^−5^	1.33379 × 10^−5^	4.46 × 10^−8^	1.79015 × 10^−8^
	50	0.0239478	0.0210904	**0.00041254**	8.76608 × 10^−5^

*f*_5_	20	0.00279802	0.00583702	2.61542 × 10^−8^	9.66738 × 10^−8^
	30	0.00294599	0.00756719	3.3219 × 10^−9^	1.53684 × 10^−8^
	50	0.0147917	0.0209218	8.75378 × 10^−6^	3.08913 × 10^−5^

The bold values denote the better value (mean or S.D.) in each line.
